# Towards more sustainable AFO manufacturing: An exploratory life cycle inventory of partially digital production

**DOI:** 10.33137/cpoj.v9i2.47795

**Published:** 2026-10-16

**Authors:** O Strenge, C Krieger, C Dilthey, L Nasilowski, J Frei, D Hochmann

**Affiliations:** 1. Biomechatronics Research Laboratory, FH Münster University of Applied Sciences, Steinfurt, Germany; 2. Kompetenzzentrum Kramer (Centre for Orthopaedic and Rehabilitation Technology), Papenburg, Germany; 3. MUUV GmbH, Münster, Germany; 4. Care Center Deutschland GmbH, Bochum, Germany

**Keywords:** Sustainability, Life Cycle Inventory, Ankle-Foot Orthosis, AFO, Digitalization, Additive Manufacturing

## Abstract

**BACKGROUND:**

Ankle-foot orthoses (AFOs) are medical devices supporting individuals with pathological gait. Manufacturing fully customized AFOs with a carbon dorsal leaf spring (C-AFOs) traditionally relies on manual processes involving material waste and resource consumption. Additive manufacturing (AM), commonly referred to as 3D printing, offers potential pathways toward a more sustainable production, yet its impact on sustainability-related metrics in orthosis manufacturing remains insufficiently examined.

**OBJECTIVE:**

In this study a partially digital C-AFO manufacturing workflow was developed using AM, initially tested and it was investigated how the partial digitalization affects selected sustainability-related metrics compared to the established manual manufacturing workflow.

**METHODOLOGY:**

An exploratory study was conducted at a German orthotic workshop, where a partially digital workflow for C-AFO manufacturing was developed and implemented. Two C-AFOs were produced using the traditional manual and the new partially digital workflow, while both workflows were analyzed and compared by means of a gate-to-gate life cycle inventory analysis in accordance with ISO 14040/44. Data collection encompassed the consumed material mass (divided into material incorporated into the C-AFO, in-house reusable material, potentially externally recyclable material, and disposable material), electrical energy consumption, and process water usage.

**RESULTS:**

Partial digitalization substantially reduced total material use by 4.59 kg (21%) and process water consumption by 12 L (83%), primarily by reducing the use of non-recyclable plaster models. Additionally, the proportions of in-house reusable and potentially externally recyclable materials increase, while the proportion of disposable material decreases. Conversely, electrical energy consumption increased by 15.96 kWh (172%), largely attributable to the 3D printing processes employed.

**CONCLUSION:**

Although the exploratory nature and limited scope of this study preclude general conclusions regarding overall workflow sustainability, the findings indicate that transitioning from manual to partially digital C-AFO manufacturing presents possible sustainability potential. A comprehensive evaluation within the framework of a complete life cycle assessment is recommended to substantiate these initial results.

## INTRODUCTION

As in almost all manufacturing sectors, additive manufacturing (AM) is steadily gaining importance in orthotic device production.^1–3^ Its implementation offers the opportunity to transform manual workflows toward digitalized assistive device production,^1,4,5^ and potentially increase customization, cost and time efficiency, and improve patient care.^1,2,4–6^

This transformation is also evident in ankle-foot orthosis (AFO) manufacturing,^7–10^ where digitalization may also enhance environmental sustainability.^2,11,12^ Identifying such potentials requires analyzing manual and digital process steps for sustainability-relevant parameters (e.g. use of resources or environmental consequences of releases). A life cycle assessment (LCA) according to ISO 14040/44, which is a method for assessing the environmental impact of products and services, is suitable for this purpose. An LCA includes a Life Cycle Inventory (LCI) in which previously defined metrics (e.g. inputs and outputs of mass or energy) are collected for each process step.^13,14^ Although LCI data does not permit statements about the entire product life cycle, it helps to identify trends and areas for further investigation.

AFOs with a carbon dorsal leaf spring (carbon DLS) are frequently used in the cooperating workshops. They are hereafter referred to as carbon AFOs (C-AFOs). Although there are analyses in literature on the manufacture of plastic AFOs,^11,12,15^ to the authors’ knowledge, no analysis of the manufacture of C-AFOs exists. A possible reason is that there is still no established digitalized C-AFO manufacturing workflow that can be implemented at the workshops participating in the study. There are approaches to digitalize C-AFO manufacturing in different workshops involving 3D scanning of the patient’s leg, digital modeling of the scan and AM of C-AFO components.^16,17^ However, these approaches are still under development and are not yet directly applicable to the cooperating workshops meaning that C-AFOs are still largely manufactured manually using traditional methods.

Traditional C-AFO manufacturing in Germany usually involves creating a negative mold of the patient’s lower leg, casting and shaping it with plaster, fabricating the individual C-AFO components based on the resulting positive model, and finishing the completed C-AFO.^18^ Here, a distinction can be made between a partially customized C-AFO with a standardized carbon DLS and a fully customized C-AFO with a customized carbon DLS.^19^ Furthermore, the manufacturing philosophies of individual German workshops differ regarding specific process steps. For example, only some of the cooperating workshops manufacture a test orthosis that enables a preliminary fit check before the C-AFO is produced, in some workshops the carbon DLS is manufactured using a prepreg process and in others using a wet lamination process.

To exploit the potential of AM, one aim of the crossworkshop ERDF/JTF (European Regional Development Fund/Just Transition Fund) research project INDiGO (Innovative nachhaltige digitale gendergerechte Orthesenversorgung), was to develop a partially digital manufacturing workflow for a fully customized C-AFO that can be directly integrated into the workshop’s daily production. In addition to potential cost and time savings, the goal of partial digitalization was to achieve greater process sustainability which is becoming increasingly interesting for the workshops from moral, environmental, and economic perspectives.

The aim of this study was to develop and initially test this partially digital manufacturing workflow and, at the same time, to examine how partial workflow digitalization affects sustainability-related parameters. Individual process steps were digitalized using AM to the extent that the process could be directly implemented at the cooperating workshops. The study is intended to assess the value of conducting a comprehensive LCA and to serve as a basis for it. Due to the diverse manufacturing philosophies, an LCI was therefore conducted as an exploratory study comparing a manual and partially digital workflow for manufacturing a single C-AFO at one workshop. Although the method presented here can also be applied to other workshops, the results cannot be generalized accordingly.

## METHODOLOGY

### GENERAL CONDITIONS

According to ISO 14040/44, the study objectives and system boundaries were established, a functional unit was defined, and all data were normalized to the functional unit.^13,14^ The study was conducted at Kompetenzzentrum Kramer (Papenburg, Germany).

As this LCI compares two in-house production workflows, only internal production steps were considered, corresponding to a gate-to-gate assessment from reception of all semi-finished products to delivery of the finished orthosis. External processes related to material and machinery manufacture, external transport, and end-of-life management, were excluded. The sustainability-relevant variables recorded were the material mass (divided into material incorporated into the C-AFO, in-house reusable material, potentially externally recyclable material, and disposable material), electrical energy, and the process water consumption.

The distinction between potentially externally recyclable and disposable material stems from the exclusion of upstream and downstream processes. Materials whose recycling could be economically viable were classified as potentially externally recyclable; those deemed uneconomical for recycling by the workshop were classified as disposable. This follows the EU Directive 2008/98/EC, which requires recycling to be assessed in terms of its technical and economic feasibility.^20^ Since material cycles had not yet been established due to the exploratory study design, each material was classified individually based on whether recycling appeared potentially economically viable, based on estimates and calculations by the workshop and authors.

As all workshop machines are electrically powered, direct CO2 emissions from combustion processes were disregarded.

Manufacturing orthoses for different patients introduces anatomical variation of each C-AFO. However, producing multiple C-AFOs for one patient is impractical. To eliminate anatomical variation, both C-AFOs were manufactured for the same subject (n = 1 per workflow). In each workflow, one C-AFO was manufactured for one of the authors (male, 95 kg, 190 cm, shoe size 45, no pathology), serving as the functional unit. To ensure comparability of both C-AFOs, materials and thicknesses in the partially digital workflow were selected oriented on the traditional manual workflow. Trim lines and shell profiles were kept consistent, and the carbon DLS were produced identically manually in both workflows. To avoid individual errors, identical production steps were performed by the same workshop employees. Both orthoses were manufactured simultaneously, minimizing experiences from one workflow influencing the other.

### STUDY PROTOCOL

Based on the manual manufacturing workflow, a partially digital workflow was developed for the workshop’s in-house C-AFO production to the extent that it could be directly implemented into the workshop’s daily production. Both workflows were monitored, with each process step documented and relevant parameters quantified and allocated to the manufactured C-AFO. Material masses were categorized, and individual process steps were grouped into distinct main process steps required for C-AFO production regardless of the workflow. Parameters within each category were summed for each main process step, enabling comparison between them. Finally, the total sums across all main process steps were calculated, and material masses of each category were summed to a total material throughput. This approach follows ISO 14040/44 LCI methodology^13,14^ and enables comparison of the workflows within the study’s scope. However, it does not permit conclusions regarding workflow sustainability. To do so, a complete LCA must be conducted.

Due to the high number of individual items, only the most relevant are presented in the following; minor items (e.g., adhesives, cleaning agents, energy for vacuum generation) were allocated to respective material masses/process steps rather than reported separately.

### WORKFLOW DESCRIPTION

#### Manual Workflow

The manual C-AFO manufacturing workflow begins with a patient assessment, during which individual anatomical and functional requirements are determined and documented on paper.

In the first production step, a plaster cast is created as a negative form of the lower leg using disposable materials (protective films, scalpels, etc.). After hardening, the cast is filled with modeling plaster, refined through targeted addition or removal of material, and dried in a circulating air heating cabinet (Model 701E7, Ottobock SE & Co. KGaA, Duderstadt, Germany).

The positive model is then used to thermoform a PETG (Polyethylene Terephthalate Glycol) test orthosis. The sheet blank is heated in an infrared sheet heating cabinet (Model 701E43, Ottobock SE & Co. KGaA, Duderstadt, Germany) to its glass transition temperature, formed over the model, and refined. Perlon and nylon stockings protect the model and ensure even vacuum distribution.

After fit approval, C-AFO production begins. The plaster positive is covered with felt, and padding and inner lining (leather and Pedilin) are secured to it. PE (Polyethylene) foot and calf shells are thermoformed over the model. The carbon DLS‘s profile is then modeled in cork on the PEcovered positive, over which a PETG sheet is thermoformed to create a negative mold. This mold is used to cast a second plaster positive, which serves as the manufacturing basis for the prepreg process. After casting, the second positive is dried in the oven described above.

Carbon prepreg mats for the carbon DLS are cut to size, placed on the model, and cured under vacuum in a heating cabinet (Model 701E8, Ottobock SE & Co. KGaA, Duderstadt, Germany), using Perlon tricots, nylon stockings, and plastic tubing as process consumables. After curing, the carbon DLS and PE shells are milled and mechanically finished. Padding, lining elements, straps, and tabs are joined by gluing, sewing, or riveting.

Finally, the orthosis is tried on by the patient and, after positive fitting assessment, handed over without packaging material.

#### Partially Digital Workflow

The partially digital workflow begins with a patient assessment, with documentation carried out entirely digitally. The patient's lower leg is then scanned using a 3D scanner (Artec Eva, Artec 3D, Senningerberg, Luxembourg), capturing the individual anatomy without physical contact. Modeling of the digital model is performed in the 3D software Geomagic Freeform (Hexagon AB, Stockholm, Sweden).

Since direct 3D printing of a test orthosis is currently not economically viable due to stringent material-specific requirements regarding transparency and post-forming capabilities, a thermoformed test orthosis is produced analogously to the manual workflow. As a physical basis, a hollow PLA (Polylactic acid) positive form is 3D printed on a Fused Deposition Modeling (FDM) printer (Bambu Lab H2D, Bambu Lab, Shenzhen, China), stabilized by filling its base with modeling plaster, the remainder with sand, and sealing it with a plaster layer. The PETG test orthosis is then thermoformed over this stabilized form and post-processed.

Once the fit is confirmed, the C-AFO shells are digitally designed in the aforementioned software and additively manufactured in PP (polypropylene) using a Formlabs Fuse 1 selective laser sintering (SLS) printer (Formlabs, Somerville, Massachusetts, USA). Due to the pilot nature of this workflow, only 6% of the powder bed’s mass was utilized. TPU (Thermoplastic polyurethane) flap and padding elements are printed on the mentioned FDM printer.

A second positive form for the prepreg process is also FDM-printed, serving the same function as the second plaster positive in the manual workflow. Since the PLA-sand-plaster form cannot withstand the high temperatures and pressures of the prepreg process, this positive is printed entirely from glass-fiber-reinforced polyamide (PA6-GF). The prepreg process then proceeds identically to the manual workflow.

After hardening, the carbon DLS is mechanically finished. Straps and inner lining (SpaceTex) are cut to size before all components are glued, sewn, or riveted in the final assembly step. The finished C-AFO is handed over directly to the patient, while the documentation happens digitally and no packaging material is required.

### MEASUREMENT METHODS AND EQUIPMENT

The sustainability-related metrics were collected as follows:

#### Material Masses

Material masses were mostly measured using a standard digital scale wherever possible (directly or via differential measurement). Otherwise, masses were calculated from manufacturer-specified weight per unit area and folding rule measurements or estimated from literature values and data sheets.

#### Electrical Energy

Electrical energy consumption was measured directly using electricity meters (supply voltage: Digital Energy Cost Meter Pro, Wentronic GmbH, Braunschweig, Germany; highvoltage: DSZ180CEE-16A MID, ELTAKO GmbH, Fellbach, Germany) where possible. If direct measurement would have interrupted production, energy consumption was estimated from average power ratings and operating times.

#### Process Water

Process water consumption was measured directly using standard 500 ml measuring cups.

Due to heterogeneous measurement methods (direct measurement, calculations or estimations), total measurement uncertainty cannot be meaningfully estimated without making additional assumptions. The resulting further inaccuracies stemming from these assumptions, in turn, are hardly meaningful to assess. Exact determination of all uncertainties would exceed the exploratory scope of this study. Thus, no absolute measurement error is specified. The parameters presented here are therefore intended solely to illustrate trends and do not represent statistically validated absolute values.

### ETHICAL APPROVAL

The study was conducted in accordance with the Declaration of Helsinki and all applicable data protection legislation. The subject was one of the authors, who provided written consent for participation and data and image publication. The documented workflows follow the workshop’s standard practice, involving no additional procedures or data collection beyond standard clinical practice. The C-AFOs were manufactured exclusively for this study and were not subsequently used as assistive devices. According to the guidelines of the Ethics Committee at FH Münster University of Applied Sciences (Steinfurt, Germany), no official ethics application was required, as no harmful effects on humans, personal data, animals, or the environment were expected.

## RESULTS

No manufacturing or printing errors occurred during the course of the study. Both C-AFOs were evaluated as fitting and being feasible by the participant and workshop staff, since no pain occurred and no pressure points or air pockets were identified. Both orthoses are 530 mm in height.

Both workflows could be divided into six main process steps (**Figure 1**). **Figure 2** shows both manufactured C-AFOs, while **Table 1** and **Table 2** provide workflow comparisons.

**Figure 1:**
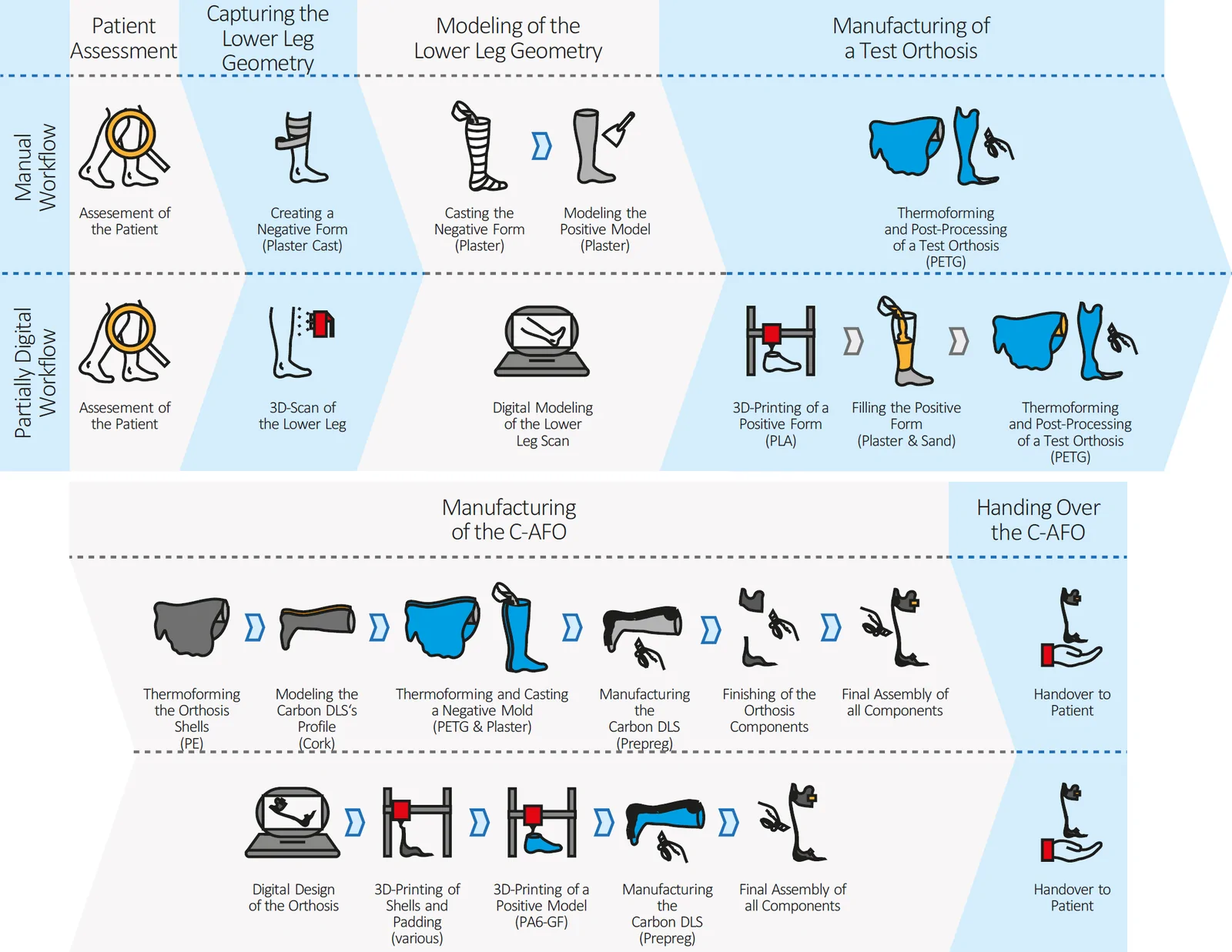
Visualization of both workflows.

**Figure 2:**
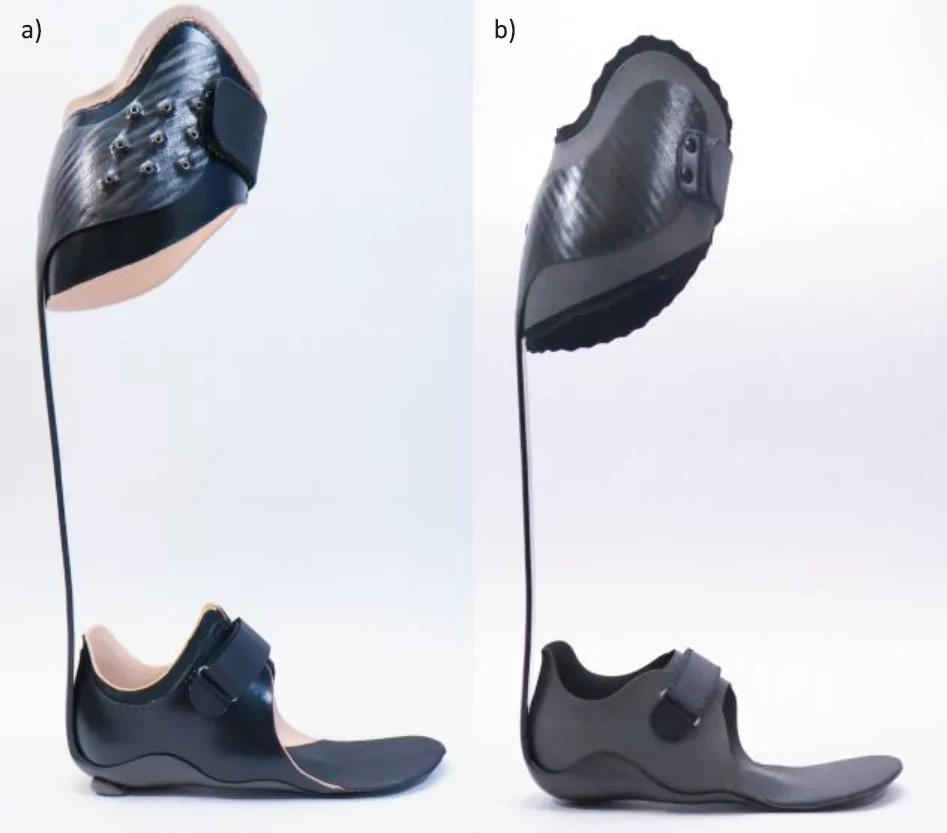
Photographs of the two manufactured C-AFOs: a) manually manufactured, b) partially digitally manufactured.

**Table 1:** Direct comparison of the examined parameters in both workflows (all values are rounded to the second decimal place); m = directly measured, c = calculated, e = estimated, Rel Share = Relative Share.

Manual Workflow					Partially Digital Workflow			
Rel. Share	Value						Value	Rel. Shar
46.1%	0.28 kg	^*m*^Carbon Dorsal Leaf Spring	**0.61 kg**	**In C-AFO incorporated Material**	**0.54 kg**	Carbon Dorsal Leaf Spring^*m*^	0.28 kg	52.3%
27.8%	0.17 kg	^*m*^Shells (PE)				Shells (PP)*m*	0.18 kg	34.3%
11.7%	0.07 kg	^*m*^Straps, Rivets, Tabs, Glue				Straps, Rivets, Tabs, Glue^*m*^	0.05 kg	9.7%
8.5%	0.05 kg	^*m*^Inner Lining (Leather)				Inner Lining (SpaceTex)^*m*^	0.02 kg	3.2%
6.0%	0.04 kg	^*m*^Padding (Pedilin)				Padding (TPU)^*m*^	>0.01 kg	0.4%
–	– –	–	**0.00 kg**	**In-house Reusable Material**	**7.23 kg**	Sand^*m*^	5.61 kg	77.4%
						Plastics (SLS Powder)^*m*^	1.63 kg	22.6%
98.6%	3.14 kg	^*m,c*^Plastics	**3.19 kg**	**Potentially Externally Recyclable Material**	**4.70 kg**	Plastics^*m,c*^	4.70 kg	100.0%
1.4%	0.05 kg	^*c*^Paper						
91.3%	16.76 kg	^*m*^Plaster	**18.36 kg**	**Disposable Material**	**5.10 kg**	Plaster*m*	4.24 kg	83.2%
2.9%	0.53 kg	^*m,e*^Disposable/Wear Parts				Test Orthosis^*m*^	0.40 kg	7.9%
2.2%	0.40 kg	Test Orthosis^*m*^				Grinding Losses^*m*^	0.25 kg	4.9%
2.1%	0.38 kg	^*m*^Grinding Losses				Disposable/Wear Parts^*m,e*^	0.15 kg	2.9%
1.6%	0.29 kg	^*m,c*^Cut-offs				Cut-offs^*m,c*^	0.06 kg	1.1%
			**9.26 kWh**	**Electrical Energy**	**25.22 kW**	3D Printing Processes^*m*^	20.39 kW	80.9%
44.2%	4.10 kW	^*m*^Drying Plaster Models				Prepreg Curing^*m*^	1.58 kW	6.3%
20.5%	1.90 kW	^*m*^Thermoforming Processes				Cutting & Grinding Processes^*m,e*^	1.47 kwh	5.8%
17.2%	1.60 kW	^*m,e*^Cutting & Grinding Processes				Digital Design & Modeling^*m,e*^	1.19 kW	4.7%
17.0%	1.58 kW	^*m*^Prepreg Curing				Thermoforming Processes^*m*^	0.54 kW	2.1%
1.1%	0.10 kW	^*m,e*^Other				3D Scanning^*e*^	0.03 kWh	0.1%
						Other^*m,e*^	0.03 kWh	0.1%
37.9%	5.50 L	^*m*^Creating Plaster Positive	**14.50 L**	**Process Water**	**2.50 L**	Filling Hollow Positive with Plaster (Test Orthosis)^*m*^	2.50 L	100.0%
34.5%	5.00 L	^*m*^Creating Prepreg Positive						
27.6%	4.00 L	^*m*^Creating Plaster Negative						

### COMPARISON OF THE PARAMETERS FOR THE INDIVIDUAL PROCESS STEPS

The LCI results for each main process step of the examined C-AFO are described below and summarized in **Table 2.**

**Table 2:** Comparison of the examined parameters for the individual process steps (all values are rounded to the second decimal place); M = manual workflow, PD = partially digital workflow, m = directly measured, c = calculated, e = estimated.

		Patient Assessment	Capturing the Lower Leg Geometr	Modeling of the Lower Leg Geometr	Manufacturing of a Test Orthosis	Manufacturing of the C-AFO	Handing Ove the C-AFO	Sum
**In C-AFO incorporated Material [kg]**	M	–	–	–	–	0.61^*m*^	–	**0.61**
	PD	–	–	–	–	0.54^*m*^	–	**0.54**
**In-house Reusable Material [kg]**	M	–	–	–	–	–	–	**0.00**
	PD	–	–	–	5.61^*m*^	1.63^*m*^	–	**7.23**
**Pot. Ext. Recyclable Material [kg]**	M	0.02^*c*^	–	–	0.67^*c*^	2.47^*m,c*^	0.02^*c*^	**3.19**
	PD	–	–	–	1.28^*m,c*^	3.42^*m*^	–	**4.70**
**Disposable Material [kg]**	M	–	0.99^*m,e*^	9.01^*m*^	0.54^*m,e*^	7.83^*m,c,e*^	–	**18.36**
	PD	–	–	–	4.78^*m,c,e*^	0.32^*m,c,e*^	–	**5.10**
**Electrical Energy [kWh]**	M	–	0.07^*e*^	1.41^*m*^	0.67^*m,e*^	7.12^*m,e*^	–	**9.26**
	PD	< 0.01^*e*^	0.03^*m*^	0.05^*e*^	2.29^*m,e*^	22.85^*m,e*^	<0.01^*e*^	**25.22**
**Process Water [L]**	M	–	4.00^*m*^	5.50^*m*^	–	5.00^*m*^	–	**14.50**
	PD	–	–	–	2.50^*m*^	–	–	**2.50**

#### Patient Assessment

Eliminating paper in the partially digital workflow slightly reduces potentially externally recyclable material mass, while the use of a tablet increases electricity consumption.

#### Capturing the Lower Leg Geometry

In the manual workflow, creating a plaster cast requires 0.99 kg of disposable material (primarily plaster bandages, plastic wraps, and tubing), 4.00 L of water and 0.07 kWh electrical energy for workstation cleaning. 3D scanning eliminates these values in the partially digital workflow, requiring only 0.03 kWh electrical energy for scanning and file conversion.

#### Modeling of the Lower Leg Geometry

Manual casting and modeling of the lower leg with plaster requires 9.01 kg of disposable material, 5.50 L of water, and 1.41 kWh of electrical energy for drying. In the partially digital workflow, these parameters are largely eliminated, with only 0.05 kWh electrical energy required for digital modeling.

#### Manufacturing of a Test Orthosis

Unlike previous steps, the manual workflow in this step shows lower consumption across all parameters, as the modeled plaster positive serves directly as the thermoforming mold. The PETG sheet cut-offs (0.67 kg) can potentially be externally recycled, while disposable materials comprise the test orthosis, which cannot be recycled due to skin contact, Perlon jerseys, and nylon stockings (0.54 kg total). Thermoforming requires 0.67 kWh electrical energy. No water is consumed.

In the partially digital workflow, the digital leg model must first be converted into a physical PLA mold, requiring modeling plaster for stabilization. The stabilizing sand (5.61 kg) can be reused in-house. Potentially externally recyclable material increases through PETG cut-offs (0.67 kg) and the 3D-printed PLA mold (0.61 kg). Modeling plaster increases disposable material to 4.78 kg and water consumption to 2.50 L, while mold printing raises electrical energy consumption to 2.29 kWh.

#### Manufacturing of the C-AFO

In contrast to the other main process steps, orthosis production shows no consistent trend across all parameters.

The manually manufactured C-AFO weighs 0.61 kg. Its production generates 2.47 kg of potentially externally recyclable material, comprising the casting mold for the second plaster positive used for the prepreg process and plastic cut-offs. This plaster positive drives disposable material mass and water consumption. Together with cut-offs and grinding losses of prepreg mats, cork, leather, padding elements, and prepreg consumables, disposable material totals 7.83 kg and process water 5.00 L. Electrical energy consumption results in 7.12 kWh, primarily for drying the plaster positive and hardening the carbon DLS.

In the partially digital workflow, the C-AFO weighs 0.54 kg, as leather inner lining was used in the manual but omitted here. The SLS printer used for printing the shells allows for a virgin PP powder refresh rate of 50%, meaning that for each print, 50% of the unsintered powder from the previous prints and 50% virgin powder can be used. Therefore 50% of the unsintered powder is reusable in-house. The remaining 50% and the FDM-printed second positive model can be potentially externally recycled. Thus, in-house reusable material amounts to 1.63 kg, and potentially externally recyclable material to 3.42 kg. Eliminating the second plaster cast reduces disposable materials to prepreg cut-offs, grinding losses, and prepreg consumables, totaling 0.32 kg. Electrical energy consumption increases to 22.85 kWh, mainly for printing the positive model (10.25 kWh), printing the shells and printer cleaning (7.41 kWh) and post-processing the shells (0.6 kWh). No water is needed.

#### Handing Over the C-AFO

As with patient assessment, digital handover negligibly reduces potentially externally recyclable material and slightly increases electrical energy consumption.

### OVERALL COMPARISON

The partially digitally manufactured orthosis weighs approximately 0.07 kg (11%) less than the manually manufactured one. Total material throughput decreases from 22.16 kg (manual) to 17.57 kg (partially digital), a reduction of 4.59 kg (21%). While the manual workflow enables no in-house material reuse, the partially digital workflow allows reuse of 7.23 kg through SLS powder and sand. Potentially externally recyclable material mass increases by 1.51 kg (47%), whereas disposable material mass and water consumption decrease by 13.26 kg (72%) and 12.00 l (83%) respectively, mainly due to reducing the amount of non-recyclable plaster models.

Electrical energy consumption increases by 15.96 kWh (172%), primarily driven by 3D printing.

## DISCUSSION

This study was conducted to develop a partially digitized C-AFO production workflow, initially implement it, and compare it with the established manual workflow in terms of sustainability-related metrics. The results show that digitalization reduces both the total material throughput and the consumption of process water, while the demand for electrical energy increases. These results suggest a potential improvement in process sustainability; however, this must be confirmed by a comprehensive LCA.

A major finding is that manual C-AFO production can be partially digitalized through AM. This alone is a positive outcome and may offer time and cost advantages, as indicated in the literature,^1,2^ independent of the results discussed below, although this requires further research.

Due to the exploratory design and gate-to-gate limitation, no general conclusions regarding sustainability can be drawn based on this LCI; however, the results can be broadly interpreted and provide a basis for further analysis.

The increase in electrical energy consumption initially appears negative. However, its environmental impact depends on the electricity mix used.^21–23^ For example, the CO₂-equivalent (CO₂e) emission factor for electricity consumed in Germany was approximately 344 g CO₂e/kWh in 2025,^24^ resulting in emissions of 3.19 kg CO₂e for the manual workflow and 8.68 kg CO₂e for the partially digital workflow. By contrast, a conservative IPCC (Intergovernmental Panel on Climate Change) estimate from 2022 reports 80 g CO_2_e/kWh for renewable energy,^25^ corresponding to 0.74 kg CO_2_e and 2.02 kg CO_2_e. Thus, potential negative effects due to increased energy demand could be reduced by renewable energy usage.

Additional efficiency gains are achievable through optimized printer utilization. The workshop’s target of powder mass utilization in established workflows is up to 25%. In this study, only 6% was used, due to the exploratory design. Since SLS energy consumption depends non-linearly on printed part volume and mass,^26^ higher powder mass utilization reduces the specific energy per printed part. To contextualize this, energy and mass consumption of an established SLS print with 24% powder mass utilization was measured, yielding 11.70 kWh (including printer cleaning). Assuming the same energy consumption for an established partially digital C-AFO manufacturing workflow with the same powder mass utilization, the shells would account for one quarter of the sintered material. Accordingly, onequarter of the total energy consumption was attributed to the shells, corresponding to 2.93 kWh. After this adjustment, the total electrical energy consumption of the partially digital workflow decreased to 20.73 kWh, representing a 124% increase compared with the manual workflow. This illustrates that establishing the partially digital workflow into daily workshop production can further reduce energy consumption. Additional reductions may be achieved by using different printing materials.

The reduction in total material throughput indicates potential for sustainability gains. Similar to the SLS energy allocation, total material throughput would decrease further due to the establishment of the partially digital workflow. Using the measurement described above, the mass of unsintered SLS powder allocated to the shells would decrease to 0.65 kg, reducing the quantities of material available for in-house reuse and potential external recycling. This would result in a total material throughput of 14.95 kg, a 33% reduction compared with the manual workflow. Furthermore, partial digitalization increases the fractions of in-house reusable and potentially externally recyclable materials, while the disposable material fraction decreases considerably. Waste production is generally associated with upstream- and disposal-related environmental impacts.^27^ Therefore, increasing in-house reusable and potentially externally recyclable material fractions may potentially offer sustainability gains, provided they are actually integrated into recycling processes. Thus, material mass impacts can only be assessed through a comprehensive LCA of an established workflow.

The same applies to the reduction in process water, which appears positive but can only be assessed by considering upstream and wastewater treatment processes.

In summary, partial digitalization of C-AFO production may offer potential for sustainability gains if two conditions are met: the use of a renewable electricity mix and the integration of reusable materials into recycling loops.^28–30^ This must nonetheless be confirmed by a comprehensive LCA.

The partially digital workflow offers further optimization potential through more efficient printer utilization and direct AM of the test orthosis. Another promising long-term approach is substituting the carbon DLS with a fully additively manufactured one, eliminating the prepreg process and associated resource consumption, though this requires development to match mechanical properties and costs.

Overall, the results are promising and demonstrate the value of conducting a comprehensive LCA of both workflows. Furthermore, the results show that fully digitalizing C-AFO manufacturing could further enhance the potentials identified here, making full digitalization a long-term goal for the manufacture of fully customized C-AFOs.

### LIMITATIONS

The participant of this study was one of the authors. As this is an exploratory study examining a single C-AFO in a single workshop for one single healthy participant, the findings of this study cannot be transferred to other workshops or scenarios, and the parameters do not permit general statements about the sustainability of manual or partially digital C-AFO manufacturing. Since only one manufacturing run per workflow was examined, no failed prints or production losses occurred and their contribution to resource and energy consumption under routine conditions, particularly in 3D printing, was not captured and should be addressed in larger studies. In addition, further studies must confirm that the two C-AFOs are functionally equivalent.

The study is further limited to a gate-to-gate LCI, excluding upstream and downstream workflows. Material classification into potentially externally recyclable and disposable categories is inherently subjective, as it depends on workshop-specific economic feasibility. Moreover, the material cycles considered ecological here must first be established and were assumed feasible only in theory.

Finally, some measurements were based on estimates, derived measurements, or manufacturer specifications, introducing uncertainties that could not be quantified.

Notwithstanding these limitations, this study provides an initial reference for future work with larger sample sizes, expanded system boundaries, and more precise measurement methods.

## CONCLUSION

This study demonstrates that partial digitalization of C-AFO production is feasible, reducing total material throughput and process water consumption by reducing the amount of non-recyclable plaster models. Disposable material mass decreases, while in-house reusable and potentially externally recyclable material fractions increase. In contrast, electrical energy demand rises due to 3D printing; however, its ecological relevance depends on the energy mix used, and renewable energy could reduce potential negative effects. Also, this study shows further optimization potential (e.g. through improved printer utilization, material substitution, and full workflow digitalization). Furthermore, this study identifies areas where improvements in the sustainability of C-AFO manufacturing due to the implementation of AM may be possible and demonstrates the value of conducting a comprehensive LCA.
